# Review on the application of imaging examination for brain injury in premature infants

**DOI:** 10.3389/fneur.2023.1100623

**Published:** 2023-02-09

**Authors:** Qing Zhang, Xihui Zhou

**Affiliations:** ^1^First Affiliated Hospital of Xi'an Jiaotong University, Xi'an Jiaotong University Health Science Center, Xi'an Jiaotong University, Xi'an, Shaanxi, China; ^2^Northwest Women's and Children's Hospital, Xi'an Jiaotong University Health Science Center, Xi'an, Shaanxi, China

**Keywords:** brain injury of premature infants, brain ultrasound, computed tomography (CT), magnetic resonance imaging (MRI), comparison

## Abstract

Brain injury is the main factor leading to the decline of the quality of life in premature infants. The clinical manifestations of such diseases are often diverse and complex, lacking obvious neurological symptoms and signs, and the disease progresses rapidly. Due to missed diagnosis, it is easy to miss the best treatment opportunity. Brain ultrasound, computed tomography (CT), magnetic resonance imaging (MRI), and other imaging methods can help clinicians diagnose and assess the type and extent of brain injury in premature infants to some extent, but the three methods have their own characteristics. This article briefly reviews the diagnostic value of these three methods for brain injury in premature infants.

## 1. Introduction

With the continuous improvement of obstetric and neonatal intensive care unit facilities, the survival rate of premature infants has increased significantly, but the complications of nerve injury (such as cerebral palsy, audiovisual dysfunction, and intellectual disability) are on the rise (1, 2). The main reason for the high incidence of nervous system damage is not yet fully developed brain function in premature infants, prone to brain injury. As the brain tissue of neonates is in the stage of continuous development and remodeling, early identification of potential sequelae of brain injury and timely intervention is of great significance to reduce complications of nervous system injury and improve prognosis (3, 4). However, the clinical manifestations of brain injury in premature infants are diverse, lacking specific neurological symptoms and signs (5), and can only be further diagnosed by imaging and other examination methods. Currently, common imaging methods include brain ultrasound, computed tomography (CT), and magnetic resonance imaging (MRI). This article reviews the diagnostic value of these three methods for brain injury in premature infants.

## 2. Definition and epidemiology of brain injury in premature infants

Brain injury in premature infants refers to pathological brain injury caused by various factors before, during, or after delivery. It is a common neurological disease in premature infants. It is not a specific term for a disease but a comprehensive concept of disease (6). Brain injury in premature infants mainly includes hemorrhagic and ischemic injuries. In clinical practice, intracranial hemorrhage (including periventricular intraventricular hemorrhage, subdural hemorrhage, and subarachnoid hemorrhage) and periventricular leukomalacia have the highest incidence. Severe brain injury may lead to neonatal visual and auditory impairment, intellectual disability, or cerebral palsy, and may even threaten the life of the newborn. According to incomplete statistics, the incidence of brain injury in premature infants in Europe and the United States is as high as 10–15% (7), whereas in China this figure is about 8%, ranking second in the world (8). The proportion of mild brain injury was about 23.48%, and the proportion of severe brain injury was about 13.57%. According to relevant research, about 25% of survivors of brain injury in premature infants will have neurological sequelae, and 10% of children will have cerebral palsy (9).

## 3. Etiology of brain injury in premature infants

The main cause of brain injury in premature infants is the sensitivity of cells and cerebrovascular to inflammatory damage caused by ischemia or infection. Because the brain of premature infants is not yet mature, the self-regulation ability of blood vessels is poor, and the unique structure and physiological characteristics of blood vessels around the ventricle (anatomically, the ventricle is in the terminal blood supply area), the brain is prone to ischemia and bleeding. Prenatal and intrapartum factors such as intrauterine infection, pregnancy-induced hypertension, and a history of abnormal delivery, as well as neonatal factors such as low birth weight, asphyxia, and shock, can cause brain damage in premature infants (10, 11).

When the white matter around the brain ventricle is ischemic, this white matter will interact with inflammatory factors, leading to the activation of microglia, leading to oxidative stress, thereby releasing pro-inflammatory cytokines, leading to glutamate toxicity, which will not only consume energy but also damage the integrity of blood vessels (12). Oligodendrocyte precursor is highly sensitive to the aforementioned factors and is vulnerable to injury during ischemia, which also affects the formation of myelin; however, intracranial hemorrhage is usually associated with the special structural vulnerability of the germinal matrix itself and cerebral blood flow fluctuations (13). The pericytes of neovascularization in the germinal stroma of premature infants are few, the substrate is immature, the end of the encapsulated astrocytes lacks glial fibrillary acidic protein, and the blood vessels grow rapidly. When cerebral blood flow fluctuates, pressure changes can easily lead to intracranial hemorrhage (14–16).

## 4. Comparison of different imaging methods in the diagnosis of brain injury in premature infants

Early diagnosis and timely intervention are important ways to improve the prognosis of brain injury and reduce the mortality rate of premature infants. However, the clinical symptoms of brain injury in premature infants are not obvious or lack characteristic identification points. To further clarify the type of lesion, it is necessary to carry out an imaging examination. At present, the imaging methods for the diagnosis of brain injury in premature infants mainly include brain ultrasound, computed tomography (CT), and magnetic resonance imaging (MRI). Each examination method has its own characteristics, so it is particularly important to understand the diagnostic value of these three imaging methods for brain injury in premature infants.

### 4.1. Diagnostic value of brain ultrasound in premature infants with brain injury

Ultrasound diagnosis is a technique that reflects human tissue structure and pathological processes through gray-scale imaging and echoes intensity changes (17). With the rapid development of ultrasonic instruments, ultrasonic diagnosis is more and more widely used in clinical practice. By the late 1970s, this technique was used to diagnose neonatal intracranial diseases. Because the fontanel of the newborn is not closed at birth, the ultrasound is not affected by the skull during the cranial ultrasound. The development of brain ultrasound technology has opened up a new way for clinicians to understand intracranial lesions *in vivo* (17). The technique has the following advantages in the diagnosis of brain injury in premature infants: ① Brain ultrasound operation is simple and fast, will not cause radiation or trauma to the children, and can be completed at the bedside, effectively avoiding the impact of multiple children exercise on the diagnosis. ② Brain ultrasound has a high resolution for central brain lesions and has specific diagnostic value for intracranial hemorrhage, posterior ventricular enlargement, hydrocephalus, and other diseases. ③ Brain injury in premature infants is a dynamic change. Brain ultrasound can display the type, location, and range of intracranial lesions in real time and provide reliable ultrasound data for clinical diagnosis. ④ It is safe and feasible to detect the hemodynamic changes of soybean stem artery in premature infants by color Doppler ultrasound. It can not only reflect the changes in local cerebral blood perfusion but can also be used to evaluate the hemodynamic changes of brain basal ganglia injury (18). ⑤ Brain ultrasound and other clinical diagnosis and treatment activities or laboratory tests do not conflict and interfere with each other, which can be carried out simultaneously to ensure the accuracy of treatment and rescue (19).

At present, the types of brain injury in premature infants diagnosed by brain ultrasound mainly include periventricular intraventricular hemorrhage and periventricular leukomalacia. The ultrasonographic manifestations of periventricular intraventricular hemorrhage in different periods are as follows (20): ① Acute hemorrhage showed a moderate strong echo, thin edge, and clear boundary; ② 2–3 days later, if there was no fresh bleeding, the local echo of the hemorrhage was uniformly enhanced; ③ 7–10 days later, blood clots into the absorption period. There was no echo in the center of the clot, and the bleeding lesions were completely absorbed or formed cysts. If 7–10 days later, the ultrasound still shows a strong echo without fading, or the brain hemisphere echo is unevenly enhanced, there are scattered rough strong echo particles, and neuronal necrosis may occur (21). Brain ultrasound diagnosis of periventricular white matter injury is usually divided into four stages (22): ① Echo enhancement stage: coronal anterior horn of the lateral ventricle, sagittal posterior triangle area, and lateral ventricle with rough, uneven, and boundary strong echo area; ② Relative normal period: mild localized echo enhancement returned to normal within a few days, and there was no abnormality in brain ultrasound images; ③ Cystic formation stage: if 1 week after the unilateral ventricle hyperechoic, it may be an incomplete recovery of the lesion. After 2–3 weeks, the original echo enhancement area may appear in size, shape, a low echo, or echoless cystic softening lesions; ④ after that, the smaller cystic softening foci become smaller or disappear after being filled with glial cells, and the larger softening foci gradually form larger cysts.

However, due to the limited depth of the probe can be detected, there is a blind area in the examination of brain surface by craniocerebral ultrasound, and the edge of the brain hemorrhage (such as subdural hemorrhage and subarachnoid hemorrhage) is not very sensitive to a missed diagnosis.

### 4.2. Diagnostic value of computed tomography (CT) for brain injury in premature infants

Computed tomography (CT) uses an X-ray beam to scan a layer of a certain thickness in a part of the human body. The X-ray passing through the layer is received by a detector and converted into visible light. The visible light is converted into electrical signals through photoelectric conversion and then converted into a digital input computer system through an analog/digital converter to obtain the final image (23). Professional doctors can obtain the corresponding focus information by interpreting the final image. This method has the advantages of a short scanning time, a clear imaging structure, and high resolution. It can not only display the pathological changes of brain tissue quickly, intuitively, and clearly but also provide clear information about the location, range, and degree of lesions. At present, it has been widely used in the diagnosis of cerebral hemorrhage, brain trauma, and other diseases (24).

Computed tomography (CT) is one of the important auxiliary tools in the early diagnosis and follow-up evaluation of brain injury in premature infants. It can not only be used to distinguish neonatal hypoxic-ischemic encephalopathy and intracranial hemorrhage (especially highly sensitive to subarachnoid hemorrhage), accurately locate the bleeding site but can also be used to diagnose periventricular leukomalacia end stage. However, according to the investigation of Yu (25), the frontal lobe tissue of the newborn is not well developed in the early stage. The white matter in computed tomography (CT) is low density, and the classification of CT is easy to make mistakes. Therefore, when relying on computed tomography (CT) to diagnose neonatal hypoxic-ischemic encephalopathy and determine prognosis, we should be cautious and need at least 1 month of follow-up review. The degree of low density (i.e., *CT*-value) and low-density morphology must be combined to objectively assess the degree of brain injury through computed tomography (CT) images. Zhang et al. (26) also confirmed this by dynamic observation of computed tomography (CT) in neonates with hypoxic-ischemic encephalopathy. They believe that in the early and middle stages of the disease, it is difficult to determine the degree of pathological changes and prognosis of neonatal hypoxic-ischemic encephalopathy by computer tomography (CT) alone. It is more valuable to evaluate prognosis and guide intervention by evaluating brain damage with CT signs at 1 month. In addition, computed tomography (CT) requires children to be fully exposed to ionizing radiation, and the examination time is long, so children need to be transported. Brenner and other scholars (27) believe that the benefits and risk exposure of using this examination to diagnose brain injury in premature infants are very unequal. Therefore, in the clinical study, computed tomography (CT) is not a routine examination method for diagnosing brain damage in premature infants. CT examination will not be performed for the time being, but the patient's condition should be closely observed. If abnormal conditions occur, a CT examination should be performed in time to improve the positive detection rate of craniocerebral injury and reduce medical costs (28).

### 4.3. Diagnostic value of magnetic resonance imaging (MRI) for brain injury in premature infants

Magnetic resonance imaging (MRI) is a relatively new medical imaging technology, which has been formally applied to clinical practice since 1982. Its appearance provides higher spatial resolution and contrast resolution for evaluating abnormal pathological changes in the brain. At present, the commonly used magnetic resonance imaging (MRI) mainly includes conventional MRI, diffusion-weighted imaging (DWI), diffusion tensor imaging (DTI), magnetic resonance spectroscopy (MRS), and magnetic sensitivity weighted imaging (SWI). Conventional MRI is an imaging technique that reconstructs images using signals generated by nuclear resonance in a strong magnetic field, including T1-weighted imaging (T1WI) and T2-weighted imaging (T2WI). This technique can clearly diagnose brain parenchymal punctate hemorrhage and parasagittal injury, that is brain injury in premature infants (29). Because it is a multi-axis examination, it can more accurately reflect the location, range, nature of intracranial lesions, and the relationship between lesions and surrounding tissues (30). However, due to the incomplete development of the brain in premature infants, T1 and T2 are prolonged in conventional MRI examinations. The white matter manifestations are the same as those of periventricular leukomalacia in the acute phase (within 1 week), both of which are low-signal T1-weighted imaging and high-signal T2-weighted imaging. Therefore, it is difficult to evaluate whether the white matter is damaged or not. The resolution of conventional MRI for fresh intracranial hemorrhage is poor (31). Therefore, the diagnostic value of conventional MRI for brain injury in early premature infants is not high (32), which is mainly used for follow-up evaluation of late premature infants. Diffusion-weighted imaging (DWI) is an imaging technique that reflects the irregular movement of water molecules in tissues using quantitative diffusion statistics (ADC). DWI is highly sensitive and specific to hypoxic-ischemic brain cells. In the early stage of hypoxic-ischemic brain damage, DWI can sensitively reflect the changes of diffusion characteristics of water molecules in brain tissue along nerve fiber bundles and across brain cell membranes (33). Therefore, in recent years, it has been widely recognized internationally that DWI screening is recommended for early brain injury in premature infants (hours to a week) (34, 35). At the same time, the decrease in ADC value can be used as an indicator to evaluate the severity of early periventricular leukomalacia (36). However, in the subacute or late stage of white matter injury, cavities or glial scar hyperplasia will occur, and the sensitivity of DWI will be seriously reduced. Diffusion tensor imaging (DTI) is a new magnetic resonance imaging technique based on DWI. Compared with DWI, DTI has added a new collection direction, which can successfully display the conduction path of nerve fibers or the circuity, direction, intersection, interruption, and destruction of nerve fiber bundles (37), and shows higher sensitivity to abnormal development of premature infants after white matter injury, especially myelination disorders (38). According to Mu et al. (39), the clinical research on DTI is limited, and its clinical application needs to be further explored. Magnetic resonance spectroscopy (MRS) is a non-invasive method for studying metabolic and biochemical indicators of living tissue, which can detect changes in chemical composition in the brain. Robertson et al. (40) found that the lactic acid/creatine (Lac/Cr), N-acetyl aspartate/creatine (NAA/Cr), myo-inositol/creatine (mI/Cr), choline/creatine (Cho/Cr), and other indexes in lateral ventricle posterior horn of premature infants with white matter damage were significantly higher than those with normal infants. Magnetic susceptibility-weighted imaging (SWI) is a three-dimensional gradient echo magnetic resonance imaging technique with a high spatial resolution developed in recent years. It can detect low-signal small-signal lesions (41). It has high sensitivity and accuracy in the diagnosis of intracranial hemorrhage in premature infants, but its application has not been popularized.

## 5. Conclusion

To sum up, brain ultrasound, computed tomography (CT), and magnetic resonance imaging (MRI) in the diagnosis of brain injury in premature infants have their own characteristics, advantages, and disadvantages. Brain ultrasound has the advantages of simple operation, low price, no radiation, no trauma, and bedside examination. Color Doppler ultrasound can also dynamically monitor the changes in cerebral blood flow, so it should be the first choice for routine screening of brain injury in premature infants (42). Computed tomography (CT) can accurately and intuitively show the location and scope of brain edema, brain tissue necrosis, intracranial hemorrhage, and other injuries, but it is easy to cause radiation in children during the examination and cannot be operated at the bedside. Magnetic resonance imaging (MRI) can image in multiple directions, has a high resolution for soft tissue, and has high diagnostic value for diseases such as punctate hemorrhage and periventricular leukomalacia. However, it is expensive and time-consuming, requires high ambient temperature, and generates large noise. Therefore, computed tomography (CT) and magnetic resonance imaging (MRI) are often used to supplement the diagnosis of brain ultrasound. The complementary application of the three imaging examinations is of great significance for the early diagnosis, treatment, and prognosis of brain injury in premature infants.

## Author contributions

QZ: manuscript drafting and idea. XZ: guidance. Both authors contributed to the article and approved the submitted version.
